# Estimating the impact of school closure on social mixing behaviour and the transmission of close contact infections in eight European countries

**DOI:** 10.1186/1471-2334-9-187

**Published:** 2009-11-27

**Authors:** Niel Hens, Girma Minalu Ayele, Nele Goeyvaerts, Marc Aerts, Joel Mossong, John W Edmunds, Philippe Beutels

**Affiliations:** 1 Interuniversity Institute for Biostatistics and Statistical Bioinformatics (I-BIOSTAT), Hasselt University, Agoralaan 1, B3590 Diepenbeek, Belgium; 2 Centre for Health Economics Research and Modeling Infectious Diseases Centre for the Evaluation of Vaccination (WHO Collaborating Centre) Vaccine & Infectious Disease Institute (VAXINFECTIO), University of Antwerp, Antwerp, Belgium; 3 Surveillance and Epidemiology of Infectious Diseases, Laboratoire National de Santé, Luxembourg, Luxembourg; 4 London School of Hygiene and Tropical Medicine, London, UK

## Abstract

**Background:**

Mathematical modelling of infectious disease is increasingly used to help guide public health policy. As directly transmitted infections, such as influenza and tuberculosis, require contact between individuals, knowledge about contact patterns is a necessary pre-requisite of accurate model predictions. Of particular interest is the potential impact of school closure as a means of controlling pandemic influenza (and potentially other pathogens).

**Methods:**

This paper uses a population-based prospective survey of mixing patterns in eight European countries to study the relative change in the basic reproduction number (R_0 _- the average number of secondary cases from a typical primary case in a fully susceptible population) on weekdays versus weekends and during regular versus holiday periods. The relative change in R_0 _during holiday periods and weekends gives an indication of the impact collective school closures (and prophylactic absenteeism) may have during a pandemic.

**Results:**

Social contact patterns differ substantially when comparing weekdays to the weekend and regular to holiday periods mainly due to the reduction in work and/or school contacts. For most countries the basic reproduction number decreases from the week to weekends and regular to holiday periods by about 21% and 17%, respectively. However for other countries no significant decrease was observed.

**Conclusion:**

We use a large-scale social contact survey in eight different European countries to gain insights in the relative change in the basic reproduction number on weekdays versus weekends and during regular versus holiday periods. The resulting estimates indicate that school closure can have a substantial impact on the spread of a newly emerging infectious disease that is transmitted via close (non sexual) contacts.

## Background

Mathematical models of how infectious diseases spread from person to person through close contacts rely on assumptions regarding the underlying transmission process. These assumptions are often summarized in the so-called 'Who Acquires Infection from Whom' matrix (WAIFW). The WAIFW matrix expresses the rate at which a susceptible individual is infected by an infectious individual and is a determinant of the basic reproduction number. Since the structure of the WAIFW matrix is both very uncertain and influential for quantitative model projections, several authors have tried to obtain direct information on social mixing behaviour using social contact surveys [1-7] or alternatively time use surveys and social network analysis [8,9]. Whereas most of these studies were based on small and unrepresentative samples, Mossong et al. [6] published the results of large and representative population based surveys on social contacts recorded on a randomly assigned day in eight European countries. Hens et al. [7] provided an in-depth analysis for one of these country surveys (Belgium), which collected information on two randomly assigned days per participant. From these studies and subsequent work, it has become clear that social contact data provide crucial information for dynamic models, aiming to simulate person to person transmission of close-contact infections [3,8,10,11] and (Melegaro, A., Jit, M., Gay, N., Zagheni, E., Edmunds, W.J. What types of contacts are important for the spread of infectious diseases? Using contact survey data to explore European mixing patterns, submitted).

In this paper, we revisit the data of Mossong et al. [6] and provide a more in depth discussion on the change in mixing behaviour from the week to weekends and regular to holiday periods by estimating the social contact matrices for the different countries for both a day during the week and a day on the weekend. If available, we also compared holiday with non-holiday ('regular') periods. Throughout this paper we define 'the week' as Monday until Sunday and 'weekday' or 'working week' as Monday until Friday. When it is stated that we compare the week with the weekend, we actually refer to an average day of the week to an average day of the weekend.

By comparing these period-specific contact matrices we estimate the associated change in basic reproduction numbers R_0_. As schools are closed during holiday and weekend periods, the relative change in R_0 _provides an indication of the impact collective school closures may have (see e.g. [12]).

Since such data were collected in each of the countries, we can study the differences in mixing behaviour between countries and assess the differential impact of holidays and weekends on 'regular' mixing and R_0_ in the various countries. This comparison would reflect the change in the way people mix at school and work since school activities are reduced to a minimum and most people do not attend work on the weekend. Additionally, for some countries, contacts were reported during either school or public holidays. We believe school holiday periods to be a better proxy for school closure than public holidays (during the (pre-summer) school holidays it is likely that most adults continue working, whereas this is unlikely on public holidays). However, the comparison between school and public holidays was not made because of small sample sizes for either of the two for several of the different countries. Still, by comparing the regular (non-holiday) and holiday periods, the impact on mixing behaviour - especially due to changes in contact behaviour for children and adolescents - can be studied. Note that during the holiday periods childcare may very well substitute school attendance for young children, implying that mixing behaviour is modified in more than one way (e.g. grandparents taking more care of children, see [7]).

In the next section, we briefly introduce the data. In the subsequent section, we introduce the regression models used to study the effects of participant characteristics on the number of contacts people make. We show how social contact matrices can be estimated and how this relates to the estimation of the next-generation operator and the basic reproduction number. Note that the reader may wish to skip this more technical part, which is non-essential to understand the remainder of the paper. In the results section we report our findings and we end with a discussion.

## Methods

### Data

A population-based prospective survey of mixing patterns in eight European countries (Belgium (BE), Great Britain (GB), Finland (FI), Germany (DE), Italy (IT), Luxemburg (LU), Poland (PL) and The Netherlands (NL)) using a common paper diary methodology was conducted as part of the POLYMOD project [6]. This study was conducted covering all age groups. A total of 7290 participants recorded characteristics of 97904 contacts during one day. The surveys were conducted between May 2005 and September 2006. A contact was defined as either a non-physical contact: a two-way conversation of three or more words in the physical presence of another person without physical contact or a physical contact: a two-way conversation with skin-to-skin touching.

Survey participants were recruited in such a way as to be broadly representative of the whole population in terms of geographical spread, age and sex. In BE, IT and LU survey participants were recruited by random digit dialling using land lines; in GB, DE and PL survey participants were recruited through a face-to-face interview; survey participants in NL and FI were recruited via population registers. Children and adolescents were deliberately oversampled, because of their important role in the spread of infectious agents. Only one person in each household was asked to participate in the study. Paper diaries were sent by mail or given face to face. Participants were explained by telephone or in person how to complete the diary. They were asked to provide contextual information about the age, sex, location and 'usual contact' frequency of each contacted person. Diaries were translated into local languages. For more information on these surveys we refer to Mossong et al. [6].

We highlight two aspects mentioned by these authors. First, contacts at work were reported differently in the different surveys due to between-country differences in survey design (see Table 1). These differences were ignored in the analyses as presented by Mossong et al. [6]. Second, the sample period for some of the countries, included at least one local holiday period (Table 1). Since schools and child care centres are typically closed during these periods, we investigate the relative impact of holiday periods on social contact patterns (see [7] for such an analysis focused on BE). Moreover we also compare contact patterns during the weekend and the week. The latter analysis could not be conducted in the regular-holiday strata because of the small sample sizes and thus warrants a marginal interpretation. In the analyses, we define a weekend to be regular when it falls in between two regular weeks and as a holiday otherwise.

**Table 1:** Details of survey methodology in each country together with school and public holiday+ periods within the sampling period ([6] and EURYDICE).

Country	BE	DE	FI	GB	IT	LU	NL	PL
Over what time period was the survey conducted?	March-May 2006	January-February, May-July 2006	March-June 2006	April-May 2006	May-June 2006	May 2005, January-March, May 2006	February-September 2006	March-April 2006

Were participants instructed not to record professional contacts (eg, with clients) in the diary?	Yes, if estimated at more than 20	Yes, if estimated at more than 10*	Yes, if estimated at more than 10	No	No	No	Yes, if estimated at more than 10	No

Maximum number of contact entries in the diary?	90	73	34	29	45	55	45	45

Holidays **	Year-**2006** Winter holidays: 27/02-03/03, Spring time holidays/Easter: 03-17/03, Public holidays: 01/05, 25/05	Year-**2006** Christmas/new year: 7 to 13 days staggered between 21/12-07/01, Winter holidays: 0 to 12 days staggered between 30/01-03/03, 3^rd ^term holidays: 0 to 11 days staggered between 22/05-17/06, Summer: 6 weeks staggered between 26/06-16/09, Public holidays: 01/01, 01/05, 25/05, 05/06, 15/06	Year-**2006** Winter holidays: 20/02-11/03, Spring time holidays/Easter:13-17/04, Summer: 03/06-mid August, Public holidays: 01/05, 25/05	Year-**2006** **1. England and Wales**: Spring time holidays/Easter: 03-14/04 or 10-21/04, **2. Scotland **Spring time holidays/Easter: 31/03-24/04 Public holidays: 01/05	Year-**2006** Public holidays: 01/05, 02/06	Year-**2005** 14-21/05 Public holidays: 01/05, 05/05, Year-**2006 **Christmas/new year: 24/12-08/01, Winter holidays:25/02-05/03, Public holidays: 01/05,25/05	Year-**2006** Winter holidays: **1. North/Central**: 18/02-26/02, **2. South **25/02-05/03, 3^rd ^term: 29/04-07/05, Summer: 01/07-03/09, Public holidays: 17/04, 30/04, 05/05, 25/05, 05/06,	Year-**2006** Spring time holidays/Easter: 13-18/04

* Note that for DE no participants recorded more than 10 professional contacts.

** Holidays with in the sampling period.

^+ ^Weekends inside or adjacent to a larger holiday period were considered as holiday period.

### Methodology

In this section the methodology used to identify the factors that influence the number of reported contacts is explained. We start from the model proposed by Mossong et al. [6] and then show how we included work contacts. We then show how the relative impact of looking at various types of contacts on the basic reproduction number can be established.

### Modelling the number of contacts

The response of interest, i.e. the participant's number of contacts within a day, is a count and a Poisson distribution seems a plausible assumption. However, the Poisson distribution assumes the equality of mean and variance, a property that is rarely fulfilled in practice. Therefore, we consider the negative binomial distribution which explicitly models overdispersion, i.e. the variance is allowed to be larger than the mean. Often, overdispersion is caused by an excess variation between response probabilities or counts, possibly originating from omitting important explanatory predictors [13]. Denote *μ *the mean parameter for the negative binomial distribution, the variance is then given by *μ *+ *αμ*^2^, where *α *≥ 0 is the overdispersion parameter. When *α *= 0, the negative binomial distribution simplifies to the Poisson distribution.

Since for some of the surveys the number of possible contact entries was limited, the number of contacts is right censored. Although we could take the country-specific censoring count, for uniformity, we opted to take the minimum of these limits, i.e. 29 contacts for the survey in GB (Table 1). To accommodate for post-stratification with respect to age and household size in each country, i.e. factors known to influence contact behaviour, we weight the individual contributions. The log-likelihood function for the weighted censored negative binomial is(i)

where *δ*_*i *_= 1 if *y*_*i *_< 29 and 0 otherwise, *u*_*i *_is the post-stratification weight of observation *i*, *y*_*i *_is the number of contacts (including work contacts) for observation *i*, *X*_*i *_is the vector of explanatory variables and *P *is the density function for the negative binomial distribution:(ii)

where *μ *= *μ*(*X*_*i*_) = exp(*X*_*i*_*β*) is the mean parameter with *β*, the vector of coefficients.

Empirical count data are frequently not only characterized by overdispersion but also excess zeros. Zero-inflated count models provide a parsimonious yet powerful way to model this type of situation. Such models assume that the data are a mixture of two separate data generation processes: one generates only zeros, and the other is either a Poisson or a negative binomial data-generating process. The result of a Bernoulli trial is used to determine which of the two processes generates an observation. A standard negative binomial model would not distinguish between these two processes, but a zero-inflated model allows for this complication. We contrasted the weighted censored negative binomial regression in (i) and (ii) with its zero-inflated version. The latter is found by replacing (ii) by(iii)

where *π *denotes the probability of the zeros-governing process and *P*(*Y *= *y*_*i*_|*X*_*i*_) denotes the negative binomial density function in (ii). Note that the covariate vector *Z*_*i *_is used to allow this probability to depend on covariates which may differ from *X*_*i*_. If *π *= 0, the zero-inflated negative binomial model simplifies to the negative binomial model. Comparing the different models can be done using the likelihood ratio test [14].

Since professional contacts were not systematically surveyed in the same way for the different countries, the aforementioned methodology cannot be applied directly. Indeed, in the diary for some countries (BE, DE, FI and NL) participants were instructed not to list their professional contacts, if the number of professional contacts was greater than 20 (for participants from BE) or greater than 10 (for participants from DE, FI and NL, see Table 1). Whereas Mossong et al. [6] used the weighted censored negative binomial model from the recorded individual contact data only, in the current paper we extend their model by taking these extra professional contacts into account, thus improving the comparability of the results between countries.

### Estimating Social Contact Matrices

In this section, we outline how the country-specific social contact matrices have been estimated. We arrange the weighted average number of counts by age classes in a "social contact matrix" *M*. Each matrix element *m*_*ij *_= E(*Y*_*ij*_) gives the mean number of contacts per day by a participant of age class *j *with persons in age class *i*. Consider the random variable *Y*_*ij*_, the number of contacts in age class *i *during one day as reported by a respondent in age class *j *(*i *= 1, ..., *I*, *j *= 1, ..., *J*), which has observed values *Y*_*ijk*_, *k *= 1, ..., *n*_*j*_, where *n*_*j *_denotes the number of participants in the contact survey belonging to age class *j*. We considered 5 year age bands. The contact rates *c*_*ij *_are related to the social contact matrix by *c*_*ij *_= *m*_*ij*_/*w*_*i*_, where *w*_*i *_denotes the country-specific population size in age class *i*, obtained from demographical data (EUROSTAT, 2006). We use a generalized linear model with negative binomial response distribution and bivariate smoothing approach [15] to estimate the number of contacts during a day in age class *i *by participants in age class *j *[6,7,10,11]. For the estimation of the matrix elements *m*_*ij*_, we take the reciprocal nature of conversational contacts into account by imposing *c*_*ij *_= *c*_*ji*_.

### Estimation of Next-Generation Matrices

Consider the next generation matrix *G *with elements *g*_*ij*_, denoting the average number of secondary infections in age class *i *through the introduction of a single infectious individual of age class *j *into a fully susceptible population. The next generation matrix determines how the risk of infection varies over age classes and is defined by(iv)

with population size *N*, mean duration of infectiousness *D *and life expectancy *L *[16]. *β *denotes the matrix of per capita rates *β*_*ij *_at which an individual of age class *i *makes effective contact, i.e. transferring the infection, with a person of age class *j*. In the literature, this matrix is often called the 'Who Acquires Infection From Whom' or WAIFW-matrix. Assuming individuals are contacted at random within age classes, we introduce a proportionality factor *q *measuring the disease-specific infectivity and susceptibility and stipulate *β*_*ij *_= *q *× *c*_*ij *_or *β *= *q *× *C*. This so-called social contact hypothesis is tenable only under the reasonable assumption that the contacts from which *C *is estimated are good proxies for those contacts responsible for disease transmission [3,10,11] and (Melegaro, A., Jit, M., Gay, N., Zagheni, E., Edmunds, W.J. What types of contacts are important for the spread of infectious diseases? Using contact survey data to explore European mixing patterns, submitted).

The basic reproduction number *R*_0 _(sometimes called basic reproductive rate or basic reproductive ratio), i.e. the mean number of secondary cases a typical single infected case will cause in a population with no immunity to the disease, is the largest eigenvalue of the next generation operator defined in (iv) [16]:(v)

*R*_0 _has threshold value 1, in the sense that an epidemic will result from introduction of the infective agent when *R*_0 _> 1, while the number of new infections per day declines right after the introduction when *R*_0 _≤ 1.

To determine the relative change in *R*_0 _from the week to weekends and from regular to holiday periods, we calculate(vi)

where indices 1 and 2 refer to the contacts registered during the weekend and week (Monday to Sunday) or holiday and regular period, respectively. It is straightforward to show that the normalizing constants cancel and thus the ratio relates only to contact data. Using a nonparametric bootstrap on the contact data by participant, 95% percentile confidence intervals for the relative change in *R*_0 _can be obtained.

## Results

We first describe the results for the number of contacts per participant and then the results for the relative change in basic reproduction number when comparing the different periods.

### Modelling the number of contacts

The results of the weighted, censored, negative binomial regression analysis using participant's age, gender, household size, day of the week, period (holiday or not) and country as explanatory variables are summarized in Table 2.

**Table 2:** Weighted censored negative binomial regression model: mean and relative number of contacts.

Category	Covariate	Number of Participants	Mean (Std Dev)	Relative no. of Reported Contacts (95% CI)
Age	< 5	660	10.21 (7.65)	1.00
	5-9	661	14.81 (10.09)	1.42 (1.27, 1.56)
	10-14	713	18.69 (13.40)	1.76 (1.58, 1.94)
	15-19	685	19.93 (21.14)	1.79 (1.61, 1.97)
	20-29	879	17.18 (25.72)	1.66 (1.51, 1.81)
	30-39	815	17.83 (21.68)	1.63 (1.49, 1.78)
	40-49	908	17.51 (23.29)	1.57 (1.43, 1.70)
	50-59	906	15.96 (20.84)	1.48 (1.35, 1.62)
	60-69	728	10.51 (14.47)	1.10 (1.00, 1.21)
	70 +	270	7.71 (10.97)	0.81 (0.73, 0.89)
	Missing*	65	10.40 (12.78)	0.94 (0.65, 1.23)
Gender	Female	3808	16.13 (21.93)	1.00
	Male	3429	15.14 (15.57)	0.97 (0.94, 1.01)
	Missing**	53	10.92 (8.60)	1.60 (1.06, 2.14)
House hold size	1	749	11.23 (18.26)	1.00
	2	1645	13.32 (17.89)	1.20 (1.13, 1.27)
	3	1683	14.67 (16.44)	1.23 (1.15, 1.31)
	4	2041	17.71 (17.67)	1.38 (1.29, 1.47)
	5	814	19.49 (29.12)	1.44 (1.34, 1.55)
	6+	358	19.30 (13.14)	1.63 (1.48, 1.79)
Day of the week	Sunday	862	11.98 (14.54)	1.00
	Monday	1032	16.36 (27.65)	1.35 (1.26, 1.45)
	Tuesday	1116	16.69 (20.16)	1.40 (1.31, 1.50)
	Wednesday	1017	16.93 (18.39)	1.40 (1.31, 1.50)
	Thursday	1069	16.86 (16.31)	1.41 (1.31, 1.51)
	Friday	1122	17.00 (18.25)	1.42 (1.33, 1.52)
	Saturday	936	12.85 (14.52)	1.19 (1.11, 1.28)
	Missing***	136	12.85 (12.26)	1.44 (1.20, 1.68)
Country	BE	750	19.30 (24.31)	1.00
	DE	1341	7.95 (6.26)	0.49 (0.46, 0.53)
	FI	1006	18.46 (32.15)	0.86 (0.80, 0.93)
	GB	1012	11.74 (7.67)	0.72 (0.67, 0.77)
	IT	849	19.77 (12.27)	1.18 (1.08, 1.27)
	LU	1051	17.46 (12.81)	1.02 (0.94, 1.09)
	NL	269	24.92 (42.70)	1.41 (1.25, 1.56)
	PL	1012	16.31 (11.45)	0.97 (0.89, 1.04)
Period	Regular	6106	16.15 (19.64)	1.00
	Holiday	1048	12.93 (16.46)	0.91 (0.86, 0.96)
	Missing***	136	12.85 (12.26)	1.09 (1.01, 1.16)

Overdispersion	alpha			0.41 (0.40, 0.43)

* Missing age was equally distributed over the other variables

** Missing gender was associated with weekday, regular period and household size 1-4.

** Missing day of the week/period was associated with DE, GB, LU and household size 2-4

The dispersion parameter was estimated at 0.41 (95% CI: (0.40, 0.43)), indicating the necessity of taking overdispersion into account. We contrasted the aforementioned model with its zero-inflated version and found that zero-inflation was non-significant (P-value 0.3173). The more parsimonious model was therefore used in further analyses.

Participants in the 10-49 years age-category had the highest number of contacts, while participants above the age of 70 years had the lowest number of contacts followed by children younger than 5 years. There was no difference in the number of contacts made between males and females. Participants living in larger households had a higher number of contacts. Participants have a greater number of contacts during the week than over the weekend, and significantly fewer contacts on Sunday during the weekend. IT and NL have a relatively high number of contacts compared to BE, LU and PL whereas DE, FI and GB have a relatively low number of contacts. The results for DE, GB, IT, LU and PL remained similar as published by Mossong et al. [6]. However inclusion of work contacts proved to be important for BE, FI and NL with a significant rise in the number of contacts made.

The differences between the sample estimates (Mean and Std Dev in Table 2) and the model-based relative number of reported contacts indicate that it is important to control for the different participant characteristics.

### Estimation of Social Contact Matrices and Relative Change in R_0_

**Figure 1:**
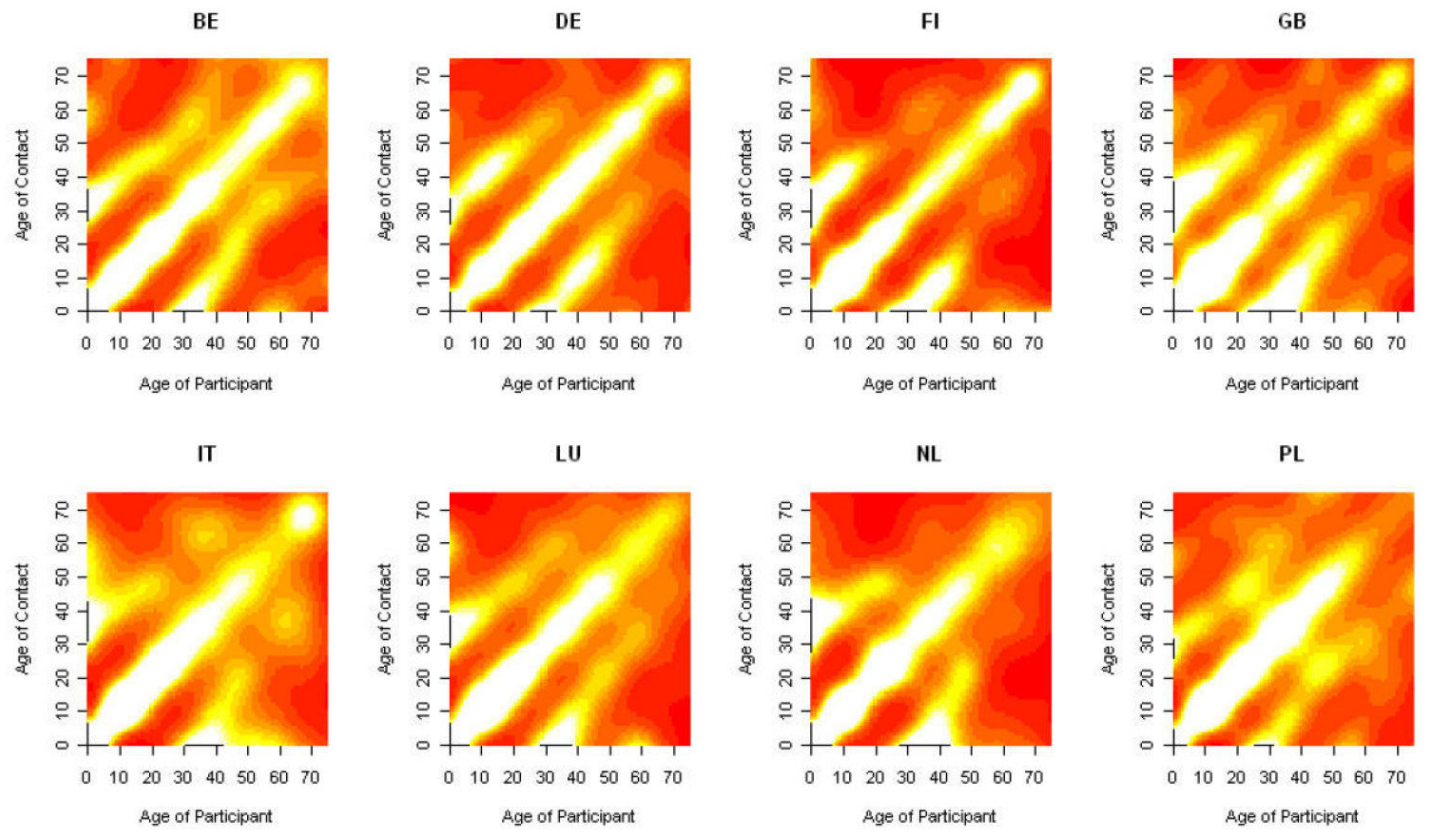
**Smoothed contact matrices**. Smoothed contact matrices for each country based on close contacts in the week weighted by sampling weights and corrected for reciprocity. White indicates high contact rates, yellow intermediate contact rates, and red low contact rates relative to the country-specific contact intensity.

A negative binomial model with bivariate smoothing approach was used to model the number of contacts per day with age class *i *made by a participant in age class *j*. We illustrate this approach for close contacts on weekdays for the eight different countries as shown in Figure 1. The country-specific patterns are very similar and show a clear assortative structure indicating people most often mix with people of similar age. The non-assortative mixing patterns originate mostly from professional contacts between people of various age-classes. The off-diagonals show mixing between age groups and can be seen to indicate social contacts between generations (e.g. in families between children-parents-grandparents).

From the estimated *M*-matrix, we derived the relative change in *R*_0 _as outlined in the methods section. The relative changes in *R*_0_, comparing the week to weekends on the one hand and regular to holiday periods on the other hand, are summarized in Tables 3 and 4, together with their 95% bootstrap-based confidence intervals based on 1000 bootstrap samples. Note that, whenever necessary, weights were adjusted to make the sample representative for the population at hand. Extra professional contacts were not taken into account in this analysis due to the shortage of additional information for these contacts. Omitting these extra work contacts has shown moderate impact on *R*_0 _since the most influential part of the contact surface determining *R*_0 _is contacts between children.

**Table 3:** Relative change in *R*_0 _from the week to the weekend for all contacts (column 3 and 4) and close contacts (column 5 and 6). '*' indicating a significant relative change in *R*_0_.

			All contacts		Close contacts	
			
Country	Number of participants in weekend vs week	Total No.	Relative Change in *R*_0_	95% Bootstrap CI.	Relative Change in *R*_0_	95% Bootstrap CI.
BE	202/544	746	0.78*	0.64, 0.94	0.88*	0.86, 0.93
DE	266/1041	1307	1.02	0.83, 1.21	1.03	0.68, 1.39
FI	283/716	999	0.78	0.73, 1.16	0.88	0.85, 1.18
GB	258/710	968	0.88*	0.69, 0.90	0.95*	0.74, 0.97
IT	226/614	840	0.80*	0.63, 0.82	0.79*	0.68, 0.99
LU	205/788	993	0.74*	0.70, 0.74	0.88*	0.66, 0.89
NL	68/189	257	0.78*	0.59, 0.79	0.79*	0.62, 0.81
PL	280/722	1002	0.77*	0.66, 0.89	0.84*	0.71, 0.86

**Table 4:** Relative change in *R*_0 _from the regular to the holiday period, all contacts (column 3 and 4) and close contacts (column 5 and 6). '*' indicating a significant relative change in *R*_0_.

			All contacts		Close contacts	
			
Country	Number of participants in Holiday vs Regular period	Total No.	Relative Change in *R*_0_	95% Bootstrap CI	Relative Change in *R*_0_	95% Bootstrap CI
BE	308/438^	746	0.83*	(0.76, 0.87)	0.90*	(0.86, 0.98)
GBˠ	371/597	968	0.87*	(0.80, 0.98)	0.83*	(0.78, 0.91)
GB†	100/868	968	0.95	(0.89, 1.17)	0.86	(0.82, 1.06)
LU	120/873	993	0.87	(0.85, 1.03)	0.90	(0.89, 1.03)
NL^ˠ^	40/217	257	0.60*	(0.56, 0.74)	0.55*	(0.49, 0.63)
NL†	39/218	257	0.60*	(0.56, 0.74)	0.55*	(0.49, 0.63)
NL†	27/230	257	0.51*	(0.49, 0.67)	0.51*	(0.46, 0.69)

^ This is a random selection of the Belgian survey, which was the only one registering two days of contacts per participant. Based on the complete Belgian survey published by Hens et al[7], the relative change in *R*_0 _was found to be 0.85, or a 15% reduction in *R*_0 _for holiday versus regular period.

ˠ Holiday period encompasses holiday periods for all regions (GB: 01/04-24/04; NL: 18/02-05/03)

† Holiday period was defined as the holiday period for one of the regions whereas the data from the other region was considered to come from a regular period (GB: 10/04-21/04; NL: 18/02-26/02 and 25/02-05/03, respectively).

Table 3 shows a significant decrease of at least 12% up to 26% in *R*_0 _due to all contacts in all countries except DE and FI, in which no significant changes in contact patterns during the weekend were recorded. For close contacts, which are believed to be better proxies for those contacts responsible for the spread of airborne infections (see [10-12]), these differences are less pronounced and the significantly lower *R*_0 _are again observed for BE, GB, IT, LU, NL and PL, ranging from 5% to 21%.

The comparison of holiday with regular periods was only possible for BE, GB, LU and NL, because only in these countries the survey was partly carried out during a holiday period. For GB and NL there were regional differences in the dates of holiday periods (Table 1). Since exact information by participant is not available, a sensitivity analysis was conducted, resulting in multiple versions of what can be interpreted as a holiday period: (1) the period encompassing all region-specific holidays (indicated by †) or (2) the holiday period of one or two of the regions only (indicated by ˠ). Although for DE holiday periods were observed, we don't wish to compare them since these periods were state-specific and scattered over the whole sampling period (Table 1). The results in Table 4 show that for BE, GBˠ and NL (NL^† ^and both NLˠ), there is a significant decrease in *R*_0 _by 17%, 13% and 40%, respectively. When focusing on close contacts, we estimate a significant decrease in *R*_0 _for BE (10%), GBˠ (17%), and NL (45%) whereas no significant difference was observed for LU.

**Figure 2:**
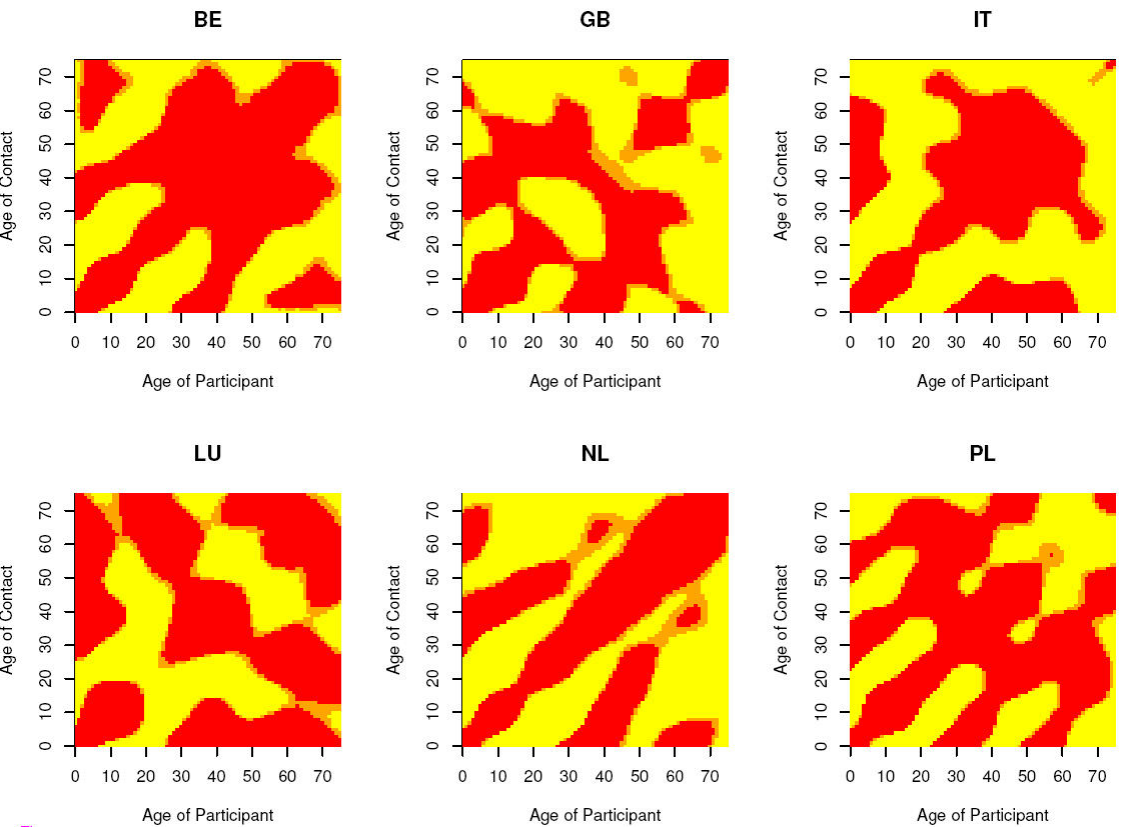
**Score matrices for the weekend to week comparison**. Matrices of scores associated to the ratios of age-specific close-contact rates when comparing weekends to the week. The scores are based on the 95% bootstrap based confidence intervals where red indicates that the ratio is significantly lower than 1 (i.e. less contacts during the weekend), orange not-significantly different from 1 (i.e. similar numbers of contacts during the week and weekends) and yellow significantly higher than 1 (i.e. more contacts during the weekend). The matrices are shown for BE, GB, IT, LU, NL and PL for which the relative reproduction number was significantly different from 1.

**Figure 3:**
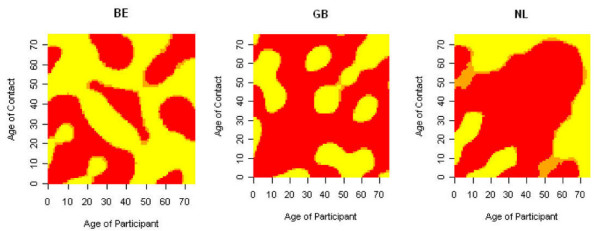
**Score matrices for the holiday to regular period comparison**. Country-specific matrices of scores associated to the ratios of age-specific close-contact rates when comparing holiday to regular periods. The scores are based on the 95% bootstrap based confidence intervals where red indicates that the ratio is significantly lower than 1 (i.e. less contacts during the holiday period), orange not-significantly different from 1 (i.e. similar numbers of contacts on regular and holiday period) and yellow significantly higher than 1 (i.e. more contacts during the holiday period). The matrices are only shown for those countries for which the relative reproduction number is significantly different from 1.

Since *R*_0 _is a summary measure of the next generation matrix and thus the contact surface, we zoom in on the relative ratios between the close contact surfaces on weekends and weekdays, and holiday and regular periods, respectively, for countries in which we observed a significant difference. We use a three-category scale based on the 95% bootstrap-based confidence intervals for the cell-specific contact ratios:(vii)

where LCL and UCL refer to the lower and upper confidence limit of the 95% bootstrap-based confidence intervals for the cell-specific contact ratios, respectively. Figure 2 and Figure 3 show the resulting score matrices.

The score matrices show greater off-diagonal mixing (less assortative) and lower (grand)parent-child components for weekends compared to the week (Monday to Sunday). That is, during the week, many contacts occur between individuals of similar age, or between parents and their children. During the weekend, more contact is made between other age groups. Clearly the rates of contact between persons of about 20-50 years are lower for weekends compared to the week due to greater professional activity during the week. A similar observation can be made when comparing holiday to regular periods although the professional contact component is less obvious for BE. Note that the red component in the score matrices is less assortative in children/adolescents for the relative ratio between holiday and regular period when compared to the relative ratio between weekend and weekdays. The result for NL relies on relatively few participants and therefore shouldn't be overinterpreted. In general, these score matrices should be interpreted with caution since sample sizes for higher age-values are small. Moreover, since scores are obtained from a pointwise comparison of the ratio and the bootstrap samples, looking at the full score surface cannot be done since multiple testing is not accounted for.

## Discussion

For a newly emerging infectious disease that is transmitted via close (non sexual) contacts, the range of prevention and control options is often limited, as specific pharmaceutical interventions (such as vaccination) are typically not (yet) available. Instead, mitigation strategies are used that focus on isolating known infectious cases, or - more generally - on reducing contacts between potentially infectious and susceptible persons. School closure is one of the strategies often considered, as children are important spreaders of many close contact pathogens, due to their frequent and intimate social contacts, their general hygiene, and perhaps their increased shedding. In this paper we assessed the impact of social distancing as a consequence of school closure and of work interruption by comparing recorded social contact behaviour during weekends and holiday periods versus the week and regular working periods, respectively. We defined a weekend to be regular when it falls in between two regular weeks and as part of a holiday period otherwise. Note that due to small sample sizes, we could not compare contact patterns between the week and the weekend in the regular/holiday strata. Therefore the results warrant a marginal interpretation.

In general, we observed a lower number of contacts during weekends compared to working weekdays (about 30% difference) and during holiday periods compared to regular periods (9% difference). We quantified the reduction in transmission by comparing the country-specific basic reproduction number for these different periods. Focusing on close contacts, believed to be most predictive for contacts enabling transmission, comparing the week to the weekends, we observed no significant difference in *R*_0 _for DE, FI and a significant decrease of 12% to 26% for BE, GB, IT, LU, NL and PL. Comparing holiday to regular periods no significant difference was observed for LU whereas a significant decrease in *R*_0 _of 10%, 17% and 45% was found for BE, GB and NL, respectively. On weekends it appears that between-generation mixing becomes more frequent (eg, through family gatherings), and same age mixing becomes relatively less frequent, particularly in BE, GB, IT, LU, NL and PL. When comparing the relative change in *R*_0 _from a working weekday (Monday-Friday) to the weekend (results not shown), we observed an even larger reduction of up to 45%. This finding again indicates a change in mixing behaviour between weekdays and the weekend and consequently the week and the weekend. During holiday periods too, BE, GB and NL show an increase in intergenerational mixing compared to the regular periods, and a decrease in same-age mixing. The Belgian data show that 25 to 35 year olds mix more frequently during holidays within their own age group (presumably because their age does not imply intense mixing in a class room type situation during a regular period, while it may imply that they spent the holidays with their friends rather than within an intergenerational family-type setting).

If we can assume that school closure in a pandemic situation resembles school closure during holiday periods, then our results show that such a strategy would have significant impact on the basic reproduction number. Similarly the additional effect of social distancing in terms of reducing work-related contacts might be observed through social contact information on weekend days. During a pandemic presumably also typical weekend activities with a strong social component such as team sports competition, and cultural outings may not take place, and therefore our estimated reductions in *R*_0 _are conservative. Similarly, typical holiday activities such as youth camps may not take place during a pandemic.

In other words, *R*_0 _potentially decreases with about 21% when considering these comparisons with weekends and holidays as proxies for school closure and associated work interruptions. Since the latter occur mostly during the weekend (and to a lesser extent during the holidays documented in the periods over which the surveys were carried out), the comparison based on holiday mixing may best approximate the impact of school closure, and the comparison based on weekend mixing may best approximate the impact of a combined school closure and work interruption strategy.

Clearly, care has to be taken when interpreting the results of this study since its design did not aim at a direct comparison of weekdays/weekends and regular/holiday periods. Using post-stratification with population-specific weights we believe we addressed this issue as much as possible. Bearing these caveats in mind, we believe that the current paper produces interesting results in that it directly uses the changes in contact patterns that occur during periods of school and/or work closure. Previous modelling studies of the potential impact of school closure for mitigating a pandemic have relied on assumptions for the reduction in contacts (see e.g. [17-19]), or have relied on assumptions for the redistribution of contacts (compensatory behaviour) [13] during periods of school closure. Several other studies estimated the impact of social distancing for the 1918 pandemic (see e.g. [20-22]) or related settings [23,24] from incidence data. We have estimated the reduction in contacts that may occur, including the compensatory behaviours. That is, our results are more driven by directly observed data than previous studies.

In summary, these results indicate that school closure would have a substantial impact for several countries whereas for some countries this would have a moderate and for one country (DE) potentially even negative impact (although non-significant here). It is noteworthy that the data collection approach in the German study (DE) digressed substantially from the other countries [6], to the extent that we believe the results based on DE to be subject to markedly more bias compared to the other countries. If transmission occurs via this route, as studies of other close-contact viruses suggest [3,10,11] and (Melegaro, A., Jit, M., Gay, N., Zagheni, E., Edmunds, W.J. What types of contacts are important for the spread of infectious diseases? Using contact survey data to explore European mixing patterns, submitted), there is potential for the emergence of complex epidemiological patterns with a decreased incidence in children partly offset by an increase in incidence in adults. A number of economic models have shown that school closure and prophylactic absenteeism have a considerable macroeconomic impact [25,26] and (Keogh-Brown, M.R., Smith, R.D., Edmunds, W.J., Beutels, P. The macroeconomic impact of pandemic influenza: estimates from models of the UK, France, Belgium and The Netherlands, submitted).

Therefore, these mitigation strategies would have to balance the effects of school closure and prophylactic absenteeism versus the macroeconomic cost of these measures.

## Conclusion

We used a large-scale social contact survey in eight different European countries to gain insights in the relative change in the basic reproduction number on weekdays versus weekends and during regular versus holiday periods. The resulting estimates indicate that school closure can have a substantial impact on the spread of a newly emerging infectious disease that is transmitted via close (non sexual) contacts.

## Competing interests

The authors declare that they have no competing interests.

## Authors' contributions

NH drafted the manuscript in consultation with PB, NG, MA, JM and JE; GMA conducted the analyses in consultation with NH, NG and MA. All authors read and approved the final manuscript.

## Pre-publication history

The pre-publication history for this paper can be accessed here:

http://www.biomedcentral.com/1471-2334/9/187/prepub
